# The Diseases of the Year 1801

**Published:** 1802-02

**Authors:** 


### The DISEASES of the YEAR 1801.

| | | | | | |
|---|---|---|---|---|---|
| Abortive and Stillborn | 407 | Deſpair | 1 | Miſcarriage | 1 |
| Abſceſs | 24 | Diabetes | 1 | Mortification | 223 |
| Aged | 1562 | Dropſy | 865 | Palpitation of the Heart | 1 |
| Ague | 4 | Eaten by Lice | 1 | Palſy | 79 |
| Apoplexy & Suddenly | 238 | Evil | 7 | Pleuriſy | 22 |
| Aſthma and Phthiſic | 427 | Fevers of all kinds | 2908 | Quinſy | 5 |
| Bedridden | 2 | Fiſtula | 3 | Rheumatiſm | 5 |
| Bleeding | 14 | Flux | 10 | Scurvy | 4 |
| Burſten and Rupture | 21 | French Pox | 14 | Small Pox | 1461 |
| Cancer | 57 | Gout | 84 | Sore Throat | 4 |
| Chicken Pox | 2 | Gravel, Stone, and Stranguary | 12 | Sores and Ulcers | 6 |
| Childbed | 170 | Grief | 3 | Spaſm | 4 |
| Colds | 6 | Headmouldſhot, Horſeſhoehead, and Water in the Head | 72 | Stoppage in the Stom. | 6 |
| Colick, Gripes, and Twiſting of the Guts | 8 | Jaundice | 79 | Surfeit | 1 |
| Conſumption | 4695 | Jaw Locked | 4 | Swelling | 3 |
| Convulſions | 3931 | Inflammation | 557 | Teeth | 333 |
| Cough, and Hooping Cough | 428 | Livergrown | 4 | Thruſh | 28 |
| Cramp | 1 | Lunatick | 127 | Vomiting and Looſeneſs | 1 |
| Croup | 14 | Meaſles | 136 | Worms | 9 |

| | | | |
|---|---|---|---|
| Chriſtened | Males - | 9400 | In all 17814 |
| | Females - | 8414 | |
| Buried - | Males - | 9661 | In all 19374 |
| | Females - | 9713 | |

Decreaſed in the Burials this Year 3694.

